# Isolating Human Monoclonal Antibodies Against Adeno-Associated Virus From Donors With Pre-existing Immunity

**DOI:** 10.3389/fimmu.2020.01135

**Published:** 2020-07-07

**Authors:** April R. Giles, Roberto Calcedo, Anna P. Tretiakova, James M. Wilson

**Affiliations:** Gene Therapy Program, Department of Medicine, Perelman School of Medicine, University of Pennsylvania, Philadelphia, PA, United States

**Keywords:** adeno-associated virus, human monoclonal antibody, memory B-cell, single cell cloning, vector engineering, gene therapy, epitope mapping

## Abstract

With the advent of single B-cell cloning technology, we can isolate antibodies against virtually any antigen to study the interaction of a given pathogen with the immune system and develop novel therapeutic strategies. Antibodies directed against the capsid of adeno-associated viruses (AAV) are a significant obstacle to effectively leveraging AAV as a gene-delivery vector in seropositive individuals. In order to design next-generation vectors that can evade neutralization by these antibodies, studies have mapped the epitopes of mouse monoclonal antibodies generated by immunization with AAV. Although these studies provide critical information regarding capsid immunogenicity, they cannot address (1) differences in the antibody repertoire generated in humans following AAV natural infection; or (2) how reactions can vary when generated in response to vector administration. Here, we isolated and evaluated a panel of novel, fully human anti-AAV antibodies by cloning single memory B cells from a seropositive normal donor. We have validated the utility of this approach to study AAV immunology. Our goal is to leverage this knowledge to design novel AAV variants that can effectively transduce target tissues in individuals with AAV-neutralizing antibodies.

## Introduction

Adeno-associated virus (AAV) is a small non-enveloped, helper virus-dependent virus that was first identified as a contaminant in a preparation of human adenovirus (1). Since then, AAV has been widely utilized in the clinic as a viral vector for many indications, culminating in the approval of Luxturna (to be delivered sub-retinally for the treatment of Leber congenital amaurosis) (2), the first AAV-based therapeutic in the United States. Shortly thereafter, Zolgensma was approved for intravenous delivery to treat type I spinal muscular atrophy (3). AAV is an ideal vector for gene therapy applications due to its lack of human pathogenicity, its inability to replicate in the absence of a helper virus, its potential for all viral genes to be replaced with a therapeutic gene of interest, and its relatively low immunogenicity profile (4–6). However, natural immunity to AAV and AAV-based vectors poses challenges to translating these vectors into therapeutics.

Humoral immune responses to AAV significantly impede the clinical use of viral vectors (7, 8). As AAV is a naturally occurring virus, many individuals become infected during the course of their lifetime. AAV seropositive individuals with neutralizing antibodies (NAbs) often cannot receive potentially life-saving therapies because NAbs can prevent the vector from reaching its target tissue, particularly in the case of intravenous vector administration (9–13). Seronegative individuals who receive therapeutic AAV vectors seroconvert following vector administration, resulting in high NAb titers that prevent vector re-administration, which may be necessary if the effect of the initial dose wanes. Therefore, the field has long sought to discover or develop AAVs with unique serological properties such that they are able to evade neutralization and transduce target tissues even in the presence of NAbs.

Initial efforts focused on identifying AAV isolates that were distinct enough from previously identified AAVs such that pre-existing NAbs did not cross-react with the novel isolates (14, 15). Although this approach led to the discovery of many new AAVs, most were either insufficiently immunologically distinct or were so structurally distinct that it altered their tissue tropism, which can occur with only a few amino-acid changes (16). In order to rationally design a novel AAV capsid that is immunologically distinct but also targets the appropriate tissue types, studies have investigated the NAb epitopes responsible for these interactions (17–19).

A common method for studying anti-AAV epitopes is generating hybridomas from mice immunized with AAV vector, followed by vector-antibody complexing and cryo-reconstruction to determine the complex structure for epitope mapping. Although this approach has provided epitope information for several clinically relevant AAV serotypes (e.g., AAV1, 2, 5, 8, and 9), these data are limited (20–24). Relatively few AAV epitopes have been mapped compared to those mapped for other viral pathogens. This could be due to the relatively low immunogenicity of the vector and the inability of hybridoma immunization protocols to produce sufficient quantities of individual antibodies (25–28). In addition, these studies may not recapitulate the circumstances of AAV exposure because repeated immunization in the presence of adjuvant in a rodent may not model either (1) an active infection with replicating AAV in the presence of the required helper virus (such as adenovirus); or (2) a single, high-dose injection of recombinant AAV vector. This latter approach is similar to vector dosing, as animals are naïve prior to vector delivery and, therefore, often generate NAbs that neutralize only the delivered serotype (29, 30). While we are not suggesting that rodent immunization does not recapitulate pre-existing immunity, the relevance of this method is unknown. Additionally, a vaccine containing a replication-deficient or attenuated virus does not always yield protective immunity that is comparable to the immunity that is generated following an active infection (31, 32). Indeed, this could be the case for AAV. Therefore, we must carefully consider our source of antibodies when using them to generate novel viral vectors.

Given the new techniques for isolating and cloning single B cells exposed to antigens in a native setting, we can now study the antibody repertoires generated in response to a variety of antigens in a high-throughput fashion. This approach has identified groups of antibodies that can neutralize pathogens such as human immunodeficiency, influenza, and dengue viruses (33, 34). Analyzing the isolated antibody sequences has led to the identification of shared features, including variable-region gene usage and complementarity-determining region (CDR) length (35–37). Moreover, the production and evaluation of these antibodies has elucidated how these viruses interact with the immune system, thereby informing vaccine design (36, 37). This approach has also identified populations of auto-antibodies and their roles in the pathogenesis of several autoimmune disorders (38, 39).

Here, we evaluated the use of this technology to isolate and study the repertoire of antibodies elicited in response to AAV natural exposure. The frequency of circulating anti-AAV memory B cells in the blood of an individual with pre-existing immunity (or vector-induced immunity) is unknown. Single B-cell cloning allows for the enrichment or selection of a specific cell population so that the native immune response to a pathogen can be studied even with a low frequency of antigen-specific cells. High-throughput studies can efficiently evaluate and compare humoral responses from different individuals, populations of individuals, or responses generated under different conditions. Thus, this is an ideal approach for studying and identifying (1) potential differences in the antibody repertoire following AAV infection or vector administration and (2) potential variations in immunodominant epitopes between individuals, types of exposure, route of administration, and vector dose.

## Materials and Methods

### Human Donor Sample Acquisition and Screening

We obtained whole blood and matched human-donor serum samples from BioIVT (previously Bioreclamation, Westbury, NY). We isolated peripheral blood mononuclear cells (PBMCs) from donor whole blood using previously described methods (40). We then froze isolated PBMCs at a cell density of 1 × 10^7^ cells/mL, which we then placed in liquid nitrogen for long-term storage. We determined NAb titers of matched donor serum against AAV2, AAV3B, AAV8, AAV9, and AAVrh10 using NAb assay as previously described (10).

### Switched Memory B-Cell Sorting

We briefly thawed 1e7 frozen donor PBMCs at 37°C, washed once, and diluted in complete media (Roswell Park Memorial Institute medium with L-glutamine, 10% fetal bovine serum or FBS), 2 mM glutamine, 10 mM HEPES, 50 g/mL gentamicin sulfate, and 1% penicillin/streptomycin) with 20 U DNase I (Cat. 776–785, Roche Scientific, Indianapolis, IN). Cells were then pelleted by centrifugation at 300 g for 5 min, followed by an additional wash in complete media with DNaseI and a final re-suspension in 10 mL complete media.

We then sorted recovered cells with the Switched Memory B-Cell Isolation Kit (Cat. 130-093-617, MACS Miltenyi Biotec, Auburn, CA). Total PBMCs were pelleted and re-suspended in re-suspension buffer [chilled phosphate-buffered saline (PBS) with 0.5% bovine serum albumin (BSA) and 2 mM EDTA]. For each kit, 100 uL of antibody cocktail (containing biotinylated α-CD2, -CD14, -CD16, -CD36, -CD43, -CD235a, -IgM, and -IgD) was added to re-suspended PBMCs and incubated with gentle agitation at 4°C for 10 min. Following cell pelleting and resuspension, 200 μL of α-biotin MicroBeads was added and cells were incubated with gentle agitation at 4°C for 15 min. Cells were then pelleted, re-suspended, and applied to an equilibrated LS column (pre-rinsed with 3 mL resuspension buffer). We then collected the flow-through, containing the enriched switched memory B-cell population. We counted cells using a hemocytometer to determine cell density.

### Validation of Memory B-Cell Population

The column fractions of interest were pelleted at 1,700 rpm for 5 min and re-suspended in the volume remaining after decanting supernatant by gentle agitation. We then stained cells with the following antibodies according to the manufacturer's protocol: α-CD19-APC (Cat. IM2470U, Beckman Coulter, Sharon Hill, PA), α-CD27-PE (Cat. 555441, Beckman Coulter, Sharon Hill, PA), and α-IgM-FITC (Cat. 555782, BD Biosciences, Franklin Lakes, NJ). Following staining, we added 2 mL staining buffer (1% FBS in PBS, sterile-filtered), pelleted the cells, and decanted and re-suspended the supernatant by agitation. We then applied 200 μL Cytofix (Cat. 554655, BD Biosciences, Franklin Lakes, NJ) to each tube and incubated for 20 min on wet ice. After adding 2 mL additional staining buffer, we pelleted cells, and decanted and re-suspended the supernatant by gentle agitation. We collected the data with a FC500 flow cytometer (Beckman Coulter, Sharon Hill, PA) and analyzed data in FlowJo (Ashland, OR, USA).

### Expansion and Irradiation of 3T3-msCD40L Cells

We acquired 3T3-msCD40L cells through the NIH AIDS Reagent Program (Cat. 12535, Germantown, MD) and thawed according to the manufacturer's protocol. Cells were cultured in Dulbecco's Modified Eagle's Medium with 10% FBS, 1% L-glutamine, and 0.1% gentamicin. Following harvest and resuspension at a density of 1e7 cell/mL, cells were irradiated with 5,000 rads (50 Gy) using an X-RAD irradiator (PXi, North Branford, CT). Irradiated cells were then cryo-preserved at a density of 1.7e7 cells/mL and stored in liquid nitrogen. This protocol was derived from (41).

### Seeding of Memory B-Cells With 3T3-msCD40L Feeder Cells

We thawed and re-suspended 2.5e8 irradiated 3T3msCD40L cells in 7.5 mL Iscove's Modified Dulbecco's Media (IMDM) with GlutaMAX (Cat. 31980030, Thermo Fisher Scientific, Waltham, MA) and 4,000 U Benzonase (EMD Millipore, now Millipore Sigma, Burlington, MA). Cells were incubated for 15 s, pelleted, and re-suspended in 10 mL IMDM with GlutaMAX.

We formulated complete media for B-cell seeding by adding 17,500 U IL-2 (Cat. 11147528001, Roche Diagnostics, Indianapolis, IN), 87.5 μg IL-21 (Cat. PHC0215, Life Technologies, Carlsbad, CA), and 1.75e8 irradiated 3T3-msCD40L cells to 1,680 mL IMDM with GlutaMAX and benzonase. Plates were seeded as follows: all outer wells of 96-well culture plates were filled with 250 μL sterile H_2_O to prevent evaporation; we added 250 μL of complete seeding media (with feeder cells) to the remaining wells in column 2 to produce supernatant for the negative control; we added 250 μL of complete seeding media with 8 cells/mL sorted memory B cells to the remaining wells to achieve a seeding density of 2 cells/well (maximum of one viable cell/well, based on viability experiments performed previously). We then incubated plates at 37°C and 5% CO_2_ for 14 days.

On day 10 after seeding, we visualized colonies of expanding B cells by microscopy. Supernatants were screened for total antibody production as early as day 12. After 14 days in culture, all supernatants were removed and stored at −80°C. The remaining cells were then lysed in 20 μL of lysis buffer (2 mL 1 M Tris-HCl, pH 8.0 with 1.7 mL RNAse inhibitor [Cat. M0314L, New England Biolabs, Ipswich, MA]) per 132 mL DEPC-treated H_2_O. We stored the plates at −80°C. This protocol was derived from (41).

### Determination of Total Antibody Concentration by Enzyme-Linked Immunosorbent Assay (ELISA)

To determine total antibody concentration in B-cell culture supernatant, high binding 96-well plates (Corning, Corning, NY) were coated overnight at 4°C with 5 μg/mL protein A (Cat. P6031, Sigma Aldrich, St. Louis, MO) in PBS. Plates were then washed 8 × with wash buffer (PBS with 0.05% Tween) and blocked with 1% BSA in PBS at room temperature for 1 h. Samples and recombinant monoclonal antibody (mAb) standard (B12 α-gp120, Cat. IT-001-b12, Immune Technology Corp., New York, NY) were applied and incubated at 37°C for 1 h followed by 8 × wash. We then repeated blocking, followed by an 8 × wash. Biotin-SP-conjugated goat α-human IgG (Cat. 109-065-098, Jackson ImmunoResearch, West Grove, PA) at 1:10,000 dilution in PBS was incubated at room temperature for 1 h followed by 8 × wash and incubation with streptavidin-HRP (Cat. ab7403, Abcam, Cambridge, United Kingdom) at 1:30,000 dilution in PBS at room temperature for 1 h. We washed the plates 8 × and developed with TMB. We quantified antibody concentration by comparing it to the concentration of recombinant B12 antibody standard.

### Screening and Selection of Anti-AAV Memory B-Cell Clones

High-binding 96-well assay plates (Corning, Corning, NY) were coated overnight at 4°C with 1e9 GC/well AAV vector (University of Pennsylvania Vector Core, Philadelphia, PA) diluted in PBS. After discarding the coating solution, we blocked plates for 2 h at room temperature with 3% BSA in PBS followed by a triple wash with wash buffer (PBS with 0.05% Tween). Next, we applied supernatant samples and incubated them for 1 h at 37°C, followed by a triple wash. Biotin-SP-conjugated goat α-human IgG (Cat. 109-065-098, Jackson ImmunoResearch, West Grove, PA) at 1:10,000 dilution in PBS was incubated at room temperature for 1 h followed by a triple wash and incubation with streptavidin-HRP (Cat. ab7403, Abcam, Cambridge, United Kingdom) at 1:30,000 in PBS at room temperature for 1 h. Plates were washed thrice and developed with TMB. We determined clones to be positive if absorbance was at least two-fold higher than the absorbance resulting from the negative control wells in column 2.

### Polymerase Chain Reaction (PCR) Amplification and Isolation of Immunoglobulin Variable Region Sequences of Positive Clones

Positive wells were thawed on ice, cell lysate was mixed by pipetting, and 4 μL of cell lysate was added to 3.5 μL of 150 ng random hexamer primer mix (Cat. 11034731001, Roche Diagnostics, Indianapolis, IN) containing 1.5% Igepal CA-630 (Cat. I8896, Sigma Aldrich, St. Louis, MO) and 5U RNAsin (Cat. N2511, Promega, Madison, WI) in nuclease-free water (Thermo Fisher Scientific, Waltham, MA) for reverse transcription. The resulting mixture was incubated at 68°C for 1 min and placed back on ice. After adding 7 μL reverse-transcription mix [15 mM dithiothreitol (Sigma Aldrich, St. Louis, MO), 5 U RNAsin (Cat. N2511, Promega, Madison, WI), 1.75 mM each dNTP (Cat. CB4420-4, Denville Scientific, Holliston, MA) 2.3 × First Strand buffer, and 50 U SuperScript III (Cat. 18080044, Thermo Fisher Scientific, Waltham, MA) in nuclease-free water], we performed reverse transcription for 5 min at 42°C, 10 min at 25°C, 60 min at 50°C, and 5 min at 94°C.

We performed immunoglobulin gene amplification as described by Wardemann et al. (42), with the modification that we used 1 μL cDNA for the first round of PCR amplification. We analyzed the second-round PCR products by agarose gel electrophoresis (1% TAE gel) to detect the presence of a 450 bp band (variable heavy chain), a 510 bp band (variable light chain, kappa), or a 405 bp band (variable light chain, lambda).

Positive bands were extracted from the gel using the QIAquick Gel Extraction Kit (Qiagen, Hilden, Germany). We cloned purified DNA using the TOPO-TA Cloning Kit (Cat. K450001, Life Technologies, Carlsbad, CA). The resulting products were transformed into TOP10 competent cells for bacterial plasmid expansion, purification, and sequencing.

### Immunoglobulin Sequence Analysis

We queried isolated sequences against the IgBLAST database (http://www.ncbi.nlm.nih.gov/igblast/) to identify the most likely germline sequence by homology. The international Immunogenetics (IMGT) default database was utilized for all searches. Total nucleotide and amino-acid substitutions from germline were determined by alignment to the most likely germline locus for each chain (excluding CDR3). We determined framework regions (FWRs) and CDR boundaries by default IgBLAST definition, which utilizes the IMGT definition of FWRs and CDRs. We determined mutation frequency by dividing the number of mutations over the length of a given sequence.

We performed total isolated-variable-sequence alignment in VectorNTI using the “Align” function. The resulting tree was used to generate **Figure 5** and identify relationships between isolated sequences.

### Recombinant Immunoglobulin Production and Evaluation

We determined the consensus amino-acid sequence for the variable chains of each B-cell clone by the Vector NTI alignment of all sequenced TOPO clones. Sequences were subsequently codon-optimized for expression in human cells by GeneArt (Thermo Fisher Scientific, Waltham, MA). We cloned paired heavy- and light-chain variable regions in an expression construct with IgG1 constant chains under the control of a CMV promoter with the PI intron and SV40 polyadenylation signal. We used a furin cleavage site/F2A linker to separate the reading frames encoding the heavy and light chains.

We produced recombinant mAbs by transfecting HEK293 cells with these constructs using polyethylenimine. Supernatants were harvested 3 days post-transfection. We used the ELISA-based quantification described above to determine mAb concentration. We determined binding to AAV2, AAV3B, AAV8, AAV9, and AAVrh10 by AAV capture ELISA (described above). We also evaluated relevant mAbs for their ability to neutralize AAV2 and/or AAV3B using the previously described NAb assay (10).

## Results

**Figure 1:**
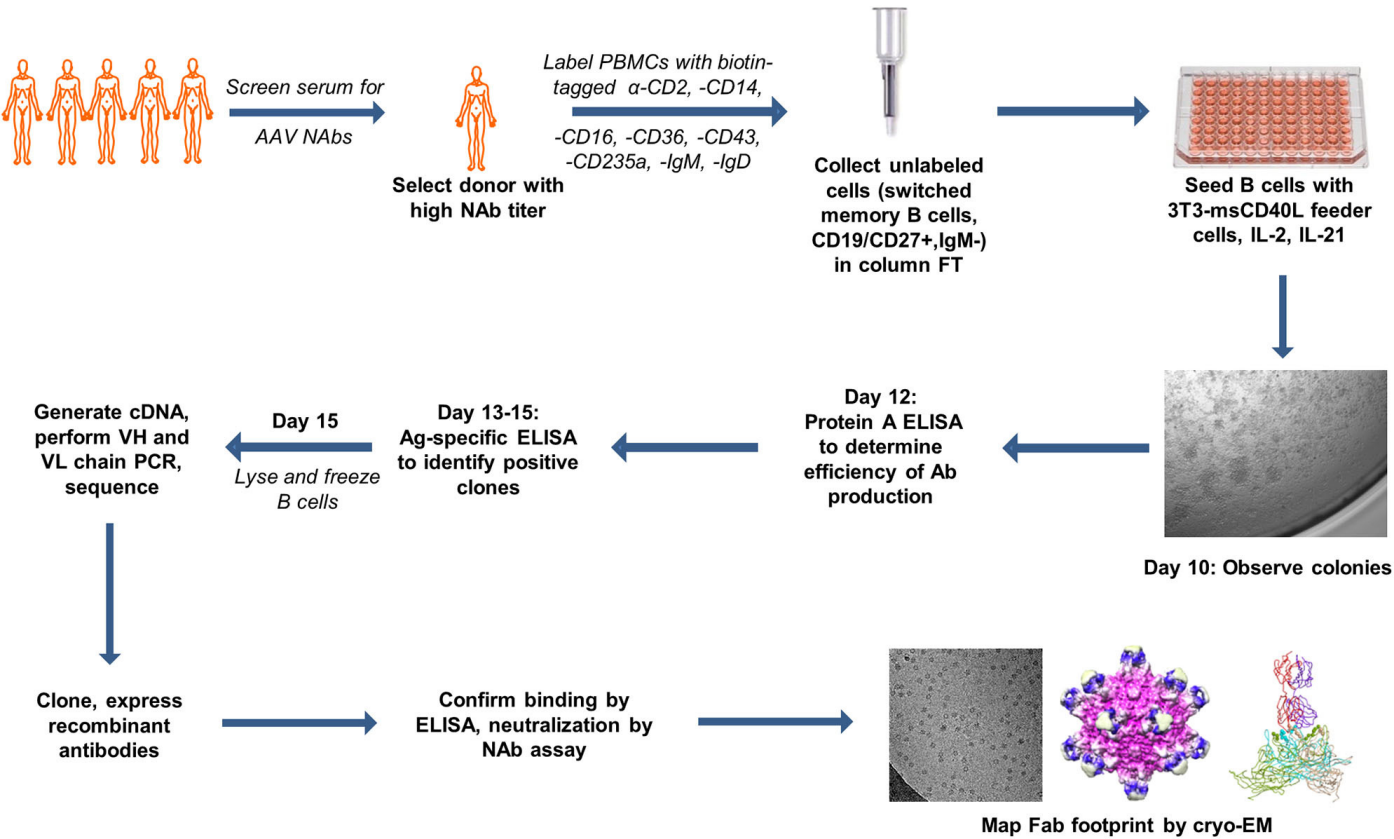
Summary of antigen-independent isolation of human donor memory B cells for the cloning of fully human anti-AAV mAbs. The figure uses motifolio image assets (www.motifolio.com). Parts of this figure (3D structures) are adapted from Giles et al. (24).

In order to select the ideal donor for memory B-cell isolation, we isolated PBMCs from the matched serum and whole blood samples of 31 normal human donors from a biorepository (Figure 1). We first screened for reactivity to AAV2, the most seroprevalent AAV serotype. Eighteen of the thirty samples (60%) were positive for NAbs against AAV2 (Table 1). This percentage of NAb^+^ samples is comparable to previously reported prevalence data of AAV2 NAbs (8, 10, 43). The NAb titers ranged from 1:5 to 1:320. We then expanded our screen to include AAV8, AAV9, and AAVrh10, and found that 11 (36%), 11 (36%), and 12 (40%) of donors, respectively, were NAb^+^ for these serotypes. The average magnitudes of these titers were lower than those observed against AAV2, suggesting cross-reactivity among NAbs, which were likely generated in response to an infection with wtAAV2.

**Table 1:** NAb titers of normal human donors against AAV.

**NAb titer**					
**Donor**	**AAV2**	**AAV3B**	**AAV8**	**AAV9**	**AAVrh10**
1	5	N/A	<5	<5	<5
2	5	N/A	<5	<5	<5
3	<5	N/A	<5	<5	<5
4	<5	N/A	<5	<5	<5
5	<5	N/A	<5	<5	<5
6	160	10	10	80	20
7	320	40	40	80	80
8	<5	N/A	<5	<5	<5
9	40	5	<5	<5	<5
10	80	10	10	20	20
11	160	40	160	1,280	160
12	<5	N/A	<5	<5	<5
13	<5	N/A	<5	<5	<5
14	<5	N/A	<5	<5	<5
15	80	N/A	5	5	10
16	80	N/A	80	80	80
17	10	N/A	<5	<5	<5
18	5	N/A	20	10	20
19	80	10	<5	<5	5
20	160	80	20	5	40
21	<5	N/A	<5	<5	<5
22	20	5	<5	<5	10
23	<5	N/A	<5	<5	<5
24	<5	N/A	<5	<5	<5
25	160	20	10	10	40
26	80	20	20	80	40
27	<5	<5	<5	<5	<5
28	<5	<5	<5	<5	<5
29	10	N/A	<5	<5	<5
30	40	N/A	10	5	20

Thirty serum samples from normal human donors were evaluated for NAb titers against AAV2, AAV3B, AAV8, AAV9, and AAVrh10.

*Titer of < (1:)5 is defined as negative (LOD of assay); N/A indicates that titer was not determined for that sample against that serotype*.

We were also interested in antibodies against AAV3B due to its potential use for intravenous, liver-directed gene therapy approaches in humans (44). We therefore determined AAV3B NAb titers in 10 donors with NAb titers against AAV2 and, as expected, individuals who were NAb^+^ for AAV2 were also NAb^+^ for AAV3B. However, despite AAV3B being closely related to AAV2, the magnitudes of these titers did not necessary correlate with each other. Based on the results of this screen, we selected donor 7 for memory B-cell isolation, as this donor had the highest NAb titer against AAV2, was NAb^+^ for all serotypes tested, and also had one of the highest titers against AAV3B.

We then employed a negative selection approach using magnetic bead sorting to obtain untouched, switched bulk memory B cells, which we defined as CD19^+^/CD27^+^/IgM^−^. In order to reserve PBMCs from donor 7 for cloning experiments, we used PBMCs from other donors to validate this approach. We determined by flow cytometry that prior to antibody labeling and column selection, 5–20% of bulk donor PBMCs were CD19^+^. The majority of these CD19^+^ B cells were CD27^−^/IgM^+^ naïve (Supplemental Figure 1). After labeling cells with a cocktail of biotinylated antibodies against non-memory B-cell markers followed by magnetic bead sorting, we determined that >90% of cells in the untouched flow-through fraction were CD19^+^. Of these cells, almost all were CD27^+^/IgM^−^, demonstrating that this population of cells is highly enriched for switched memory B cells. We conclude that this process efficiently sorted memory B cells, as only a small fraction of the CD19^+^ cells retained on the column were CD27^+^/IgM^−^.

To culture the sorted memory B cells, we diluted the bulk cells in IMDM^+^ (GlutaMAX™) media to a concentration that seeds no more than one viable cell per well, which we calculated based on prior viability determination experiments. To promote memory B-cell survival, expansion, and growth, we supplemented the media with IL-2 and IL-21. Additionally, we co-seeded these cells with irradiated 3T3-msCD40L feeder cells to provide CD40 receptor engagement. In total, we seeded 10,800 total cells into 5,400 wells, assuming a maximum viability of 50% following seeding.

Prior to harvesting supernatant, lysing cells, and storing for future RNA lysis at day 14 post-seeding, we sampled a subset of supernatants by Protein A ELISA to determine viability and antibody secretion. We found that 33% of wells sampled contained supernatant with detectable secreted antibody, indicating that one in three wells contained an expanded B-cell population from at least one seeded memory B cell. Assuming a seeding density of two cells per well, this indicates that total cell viability may have been as low as 16.5%.

We screened all supernatants (5,400 total samples) for reactivity to AAV2 and AAV3B by ELISA. These screens identified 61 supernatants that were positive for antibodies against AAV2 and 60 supernatants that were positive for antibodies against AAV3B. We found 21 supernatants that bound to both AAV2 and AAV3B; therefore, 39 supernatants were positive for AAV2 alone, 40 were positive for AAV3B alone, and 21 were cross-reactive. Based on an estimated viability between 16.5 and 33%, and assuming 101 unique positive hits, we estimated that the frequency of AAV-specific memory B cells in this normal human donor was between 2.8 and 5.5%.

Based on the magnitude of supernatant binding to capsid (either AAV2 or AAV3B) we selected colonies from which to harvest RNA, produce cDNA, and clone variable heavy and variable light chain sequences by nested PCR. We obtained and cloned VH sequences and at least one cognate chain (and in some cases, more than one light chain sequence) from 14 positive wells into an expression cassette. For this, we utilized IgG1 constant heavy and light chains to produce recombinant mAbs for further evaluation (validated sequences; Table 2). We obtained sequences from 15 additional wells but were unable to do so in a paired fashion. This was either due to an inability to isolate the sequence of one of the chains or, in the case of well 8F9, the isolation of too many sequences to efficiently evaluate all paired combinations (non-validated sequences).

**Table 2:** List of isolated anti-AAV antibody sequences and corresponding IMGT germline loci.

**Clone ID**	**Heavy**	**Kappa**	**Lambda**
1G4	4-59*08	3-20*01	
8F9	3-33*03	4-1*01	
	3-48*01		
	4-30-4*01		
	1-46*01		
15G3	1-46*01	4-1*01	
21B6	3-33*03		2-11*01
22C8	1-18*01		
26F4	3-49*04	2-28*01	
30D5	1-2*02		2-14*01
31C3	4-61*02	4-1*01	
46C11	3-48*01	1-13*02	
46D10	3-30*02	1-5*03	
46F4	4-39*07	2-29*02	
47D11	3-30*02	3-11*01	
51B6	3-21*03		1-40*01
53C10*	3-49*04	3-11*01	1-47*01
55B4	3-48*01	1-5*01	
65F3	3-9*01		1-40*01
72D3	3-30*02	3-11*01	
74E4	4-30-4*01	3-11*01	
75B3	1-18*01		
77B10	3-33*03		2-11*01
	4-59*08^∧^		
81C10	3-30*02		
81G5	4-39*01		1-40*01
86D7	3-30*09		2-11*01
92G6*	3-9*01	1-13*02 (c3)	2-11*01
	3-30*02^∧^	3-11*01(c9)	
92G8	3-49*04		
98C6	3-48*01		2-14*01
99E8	4-61*02		
100E4	3-30*04		3-9*01
100G3	4-61*02		3-1*01

Underlined clone IDs were produced recombinantly and evaluated in vitro by ELISA and NAb assay. *= all combinations of heavy and light chains were evaluated, ^∧^= this chain was excluded from evaluation.

**Figure 2:**
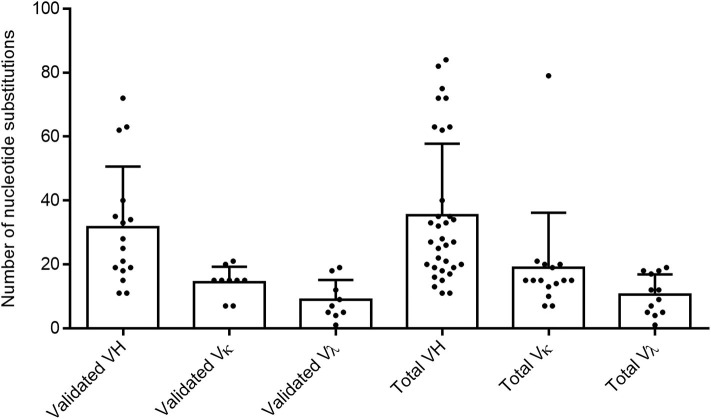
Evaluating total nucleotide mutations in AAV memory B-cell variable region sequences. Average total nucleotide substitutions from the most likely germline allele (determined by IMGT) were quantified for variable heavy (H), variable kappa (κ), and variable lambda (λ) chain sequences of anti-AAV mAbs confirmed to bind AAV *in vitro* (“validated”) or of all sequences isolated from cultures with AAV-reactive supernatant (“total”). Data are reported as mean + SD.

To confirm the memory B-cell phenotype of our isolated cells, identify the immunoglobulin germline gene usage, and evaluate the degree of maturation of each clone, we analyzed the variable heavy and variable light chain sequences from both validated and non-validated AAV-positive wells. A hallmark of memory B-cell IgG sequences is affinity maturation by somatic hypermutation (45). This process introduces nucleotide substitutions into the variable regions of immunoglobulin genes in B cells in an attempt to further improve antigen recognition and antibody functionality. We used the IMGT-based IgBLAST to map our variable chain sequences to their germline sequence, define the boundaries of the three FWRs and the first two CDRs, and quantify the number of nucleotide and amino acid substitutions from the germline. With the validated antibody sequences, we found that the VH, VK, and Vλ genes harbored an average of 31.6, 14.4, and 8.9 nucleotide substitutions, respectively (Figure 2). When we included both the validated and non-validated sequences, the averages were slightly higher, with an average of 35.5, 19, and 10.6 nucleotide substitutions for VH, VK, and Vλ genes, respectively; however, these differences were not significant.

**Figure 3:**
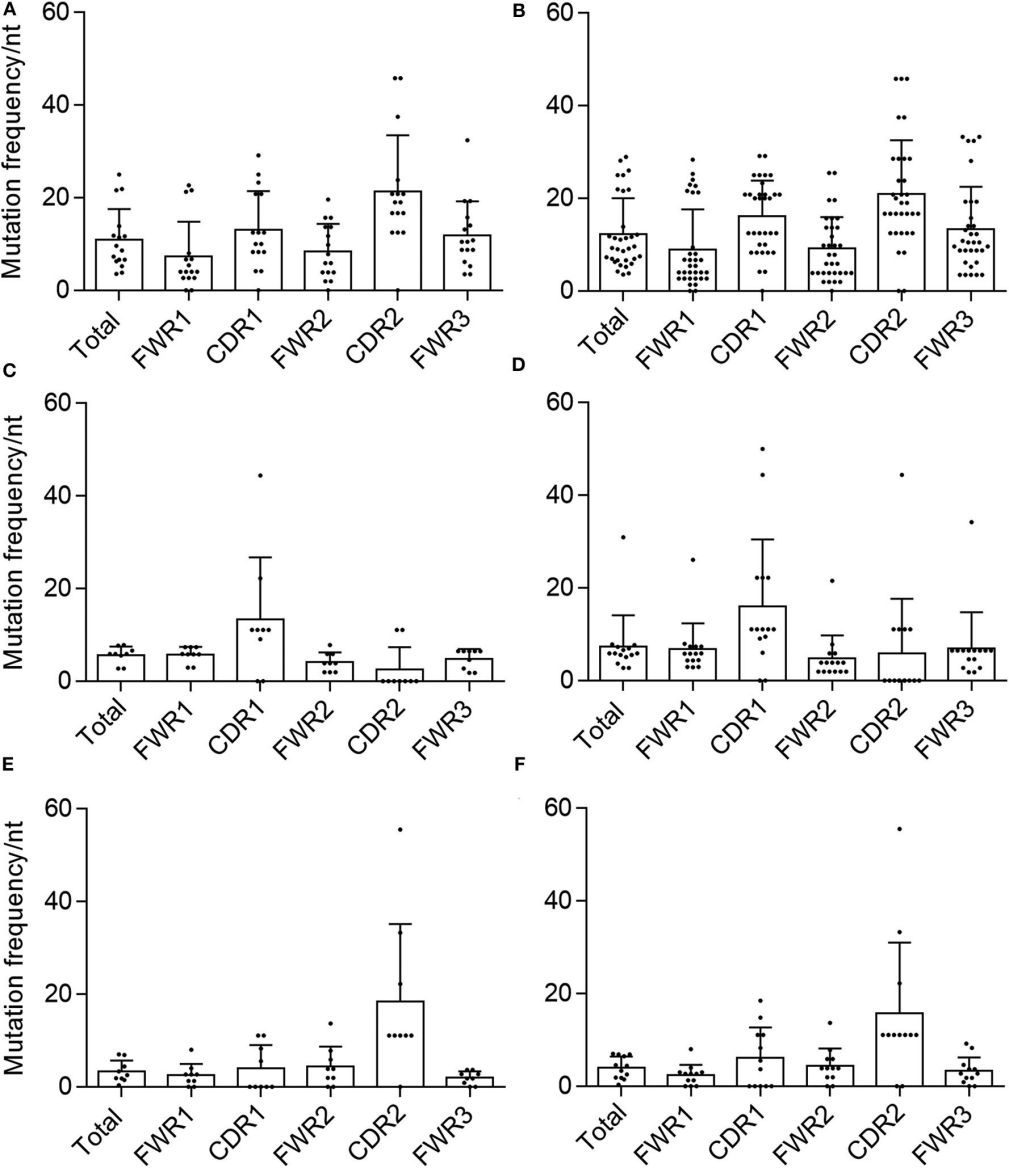
Mutational frequency in AAV memory B cell-isolated variable chain sequences. We determined the frequency of nucleotide (nt) substitutions, defined as the percentage of positions containing a substitution, in the total isolated sequence (“total”), framework regions (“FWR1,” “FWR2,” “FWR3”), and complementarity determining regions (“CDR1,” “CDR2”) (as defined by IMGT). Frequencies were determined for validated **(A)** variable heavy, **(C)** variable kappa, and **(E)** variable lambda sequences as well as all isolated **(B)** variable heavy, **(D)** variable kappa, and **(F)** variable lambda sequences. Data are displayed as mean ± SD.

We then calculated the mutation frequency for each chain based on the total number of nucleotide substitutions and the length of the predicted germline sequence (excluding CDR3). We found that the average mutation frequencies for validated sequences were 10.9, 5.7, and 3.3% for VH, VK, and Vλ, respectively (Figure 3). After including the non-validated sequences to determine total mutation frequencies, the average mutation frequency per nucleotide was 12.3% for VH, 7.4% for VK, and 4% for Vλ, which are slightly but not significantly higher than the averages obtained for the validated sequences alone.

As expected, these frequencies were orders of magnitude above the average rate of genome somatic mutations. Both the average quantity of mutations per chain as well as the average mutation frequency were in agreement with previously published values for other characterized CD27^+^/IgG^+^ memory B-cell populations (27, 46–51).

Another hallmark of memory B-cell VH and VL sequences is the concentration of mutations in the CDRs over the FWRs, likely due to the fact that the CDRs are thought to be largely responsible for antigenic interactions (28, 47). We therefore determined the average mutation frequency for each of FWRs 1–3 and CDRs 1 and 2. In both validated and total VH and Vλ sequences, the average mutation frequencies in CDR1 and CDR2 were higher than any FWR, and the mean mutation frequency for the total chain sequence, with the exception of Vλ-CDR1 and Vλ-FWR2 (Figure 3). This was not the case for the VK samples. For both the validated and total sequences, Vk-CDR1 had a higher frequency of mutations than any FWR or the total average, whereas VK-CDR2 did not. This was largely due to a number of VK sequences that were completely devoid of mutations in CDR2.

**Figure 4:**
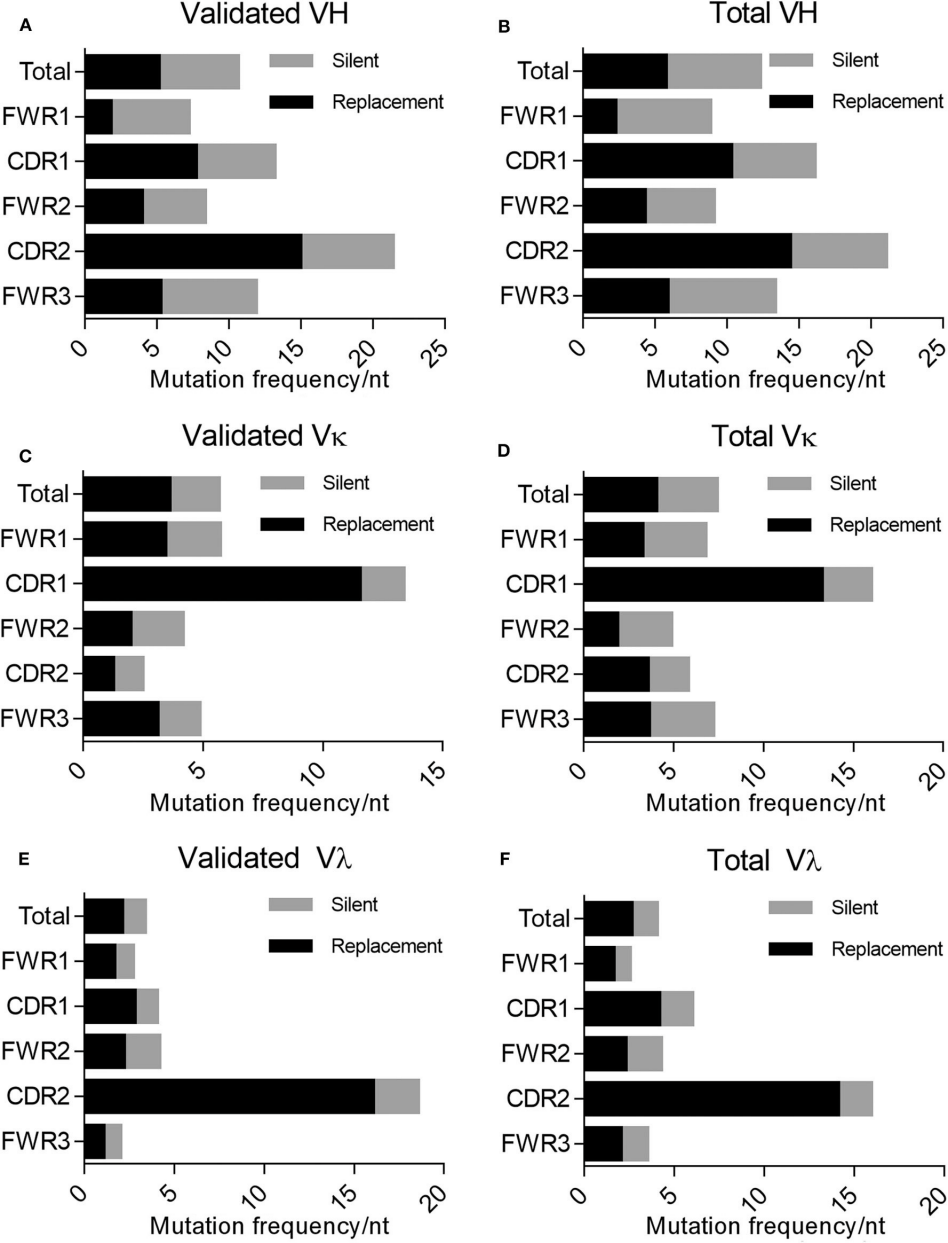
Evaluating silent vs. replacement mutational frequency in AAV memory B cell-isolated variable chain sequences. Frequency of nucleotide substitutions resulting in amino acid substitutions (R, replacement) and frequency of nucleotide substitutions not resulting in amino acid substitutions (S, silent) were quantified (based on IMGT best germline DNA/protein sequences) for the complete isolated sequence (“total”), framework regions (“FWR1,” “FWR2,” “FWR3”), and complementarity determining regions (“CDR1,” “CDR2”). Frequencies were determined for validated **(A)** variable heavy, **(C)** variable kappa, and **(E)** variable lambda sequences as well as all isolated **(B)** variable heavy, **(D)** variable kappa, and **(F)** variable lambda sequences. R/S ratios were also calculated based on these frequencies; values are indicated to the right of bars. Data are displayed as means. We determined the frequency of nucleotide (nt) substitutions, defined as the percentage of positions containing a substitution.

In addition to mutations being enriched in the CDRs relative to the FWRs, the percentage of these mutations resulting in a replacement mutation rather than a silent mutation is also higher in the CDRs of the immunoglobulin loci in memory B cells; this phenomenon is defined by the R/S ratio, or the number of replacement mutations occurring per silent mutation, with a value over 1 being indicative of a preference for a replacement mutation. We determined the R/S ratio for the aforementioned variable chain sequences (Figure 4). In the validated sequences, the overall R/S ratio was 0.9 for VH, 1.7 for VK, and 1.7 for Vλ (Table 3). When evaluating all isolated sequences, the R/S ratios were similar at 0.9, 1.2, and 1.9 for total VH, VK, and Vλ, respectively. We then evaluated the R/S ratios for the CDRs and the FWRs individually. FWR R/S ratios ranged from 0.32 (VH-FWR1, validated) to 1.76 (VK-FWR3, validated), but most ratios were around 1. The R/S ratios for the CDRs, however, ranged from 1 (VK-CDR2) to 6.5 (Vλ-CDR2), indicating that all CDRs favored replacement mutations. These values trended toward being lower than published averages; however, the CDR R/S was higher than the FWR R/S, indicating a memory B-cell phenotype, and the ratio of CDR R/S: FWR R/S was similar to published values (49, 52–55).

**Table 3:** Summary of R/S ratios of variable region sequences from AAV memory B cells.

**Chain**	**R/S**					
**VH**	**Total**	**FWR1**	**CDR2**	**FWR2**	**CDR2**	**FWR3**
Validated	0.93	0.32	1.52	0.89	2.33	0.79
Total	0.87	0.34	1.81	0.89	2.18	0.79
**Vκ**	**Total**	**FWR1**	**CDR2**	**FWR2**	**CDR2**	**FWR3**
Validated	1.74	1.50	5.75	0.90	1.00	1.76
Total	1.21	0.92	4.27	0.61	1.67	1.02
**Vλ**	**Total**	**FWR1**	**CDR2**	**FWR2**	**CDR2**	**FWR3**
Validated	1.67	1.57	2.00	1.11	6.50	1.11
Total	1.89	1.75	2.17	1.17	7.50	1.42

**Figure 5:**
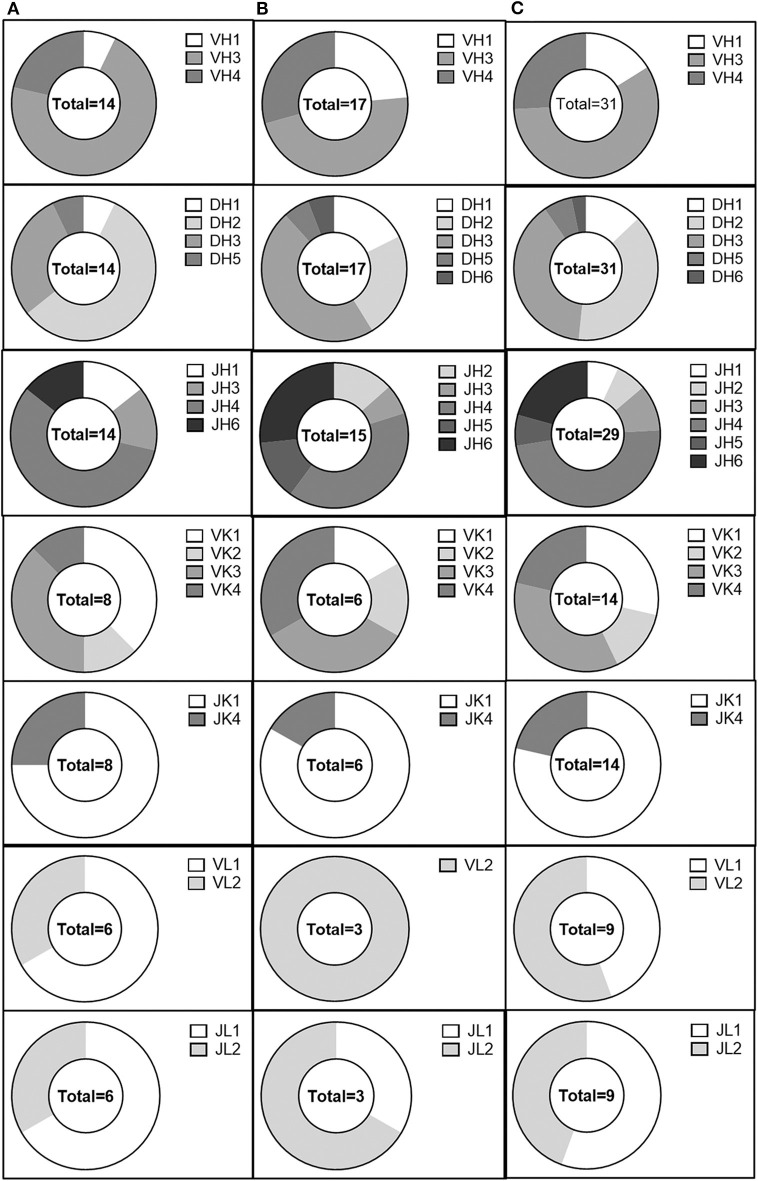
Analysis of the AAV memory B-cell IgG gene repertoire. IgH V, D, and J, IgK V, and J, and IgL V and J gene distribution in **(A)** validated, **(B)** non-validated, and **(C)** total cloned sequences.

We then examined the germline immunoglobulin gene family usage for evidence of skewing within our population of anti-AAV antibodies in the validated, non-validated, and combined antibody populations (Figure 5). We did not observe any profound differences between the usage distribution in our population and previously published distributions (48). However, our sample size was limited and may not have been large enough to distinguish such trends. Within our limited population, we also looked for evidence of enrichment of specific germline allele usage within each gene family, both for heavy and light chains (Supplemental Figures 2, 3). IGHV3-30^*^02, IGHV3-33^*^03, IGHV3-48^*^01, IGHV3-49^*^04, and IGHV4-61^*^02 were utilized by at least three separate clones. Despite the small sample size, we also observed the use of IGKV3-11^*^01 and IGKV4-1^*^01 for at least three kappa-utilizing clones and the use of IGLV1-40^*^01 and IGLV2-11^*^01 for at least three lambda-utilizing clones. These data suggest an enrichment for B-cell lineages utilizing these alleles; however, the sample size limits the conclusions that can be drawn.

**Figure 6:**
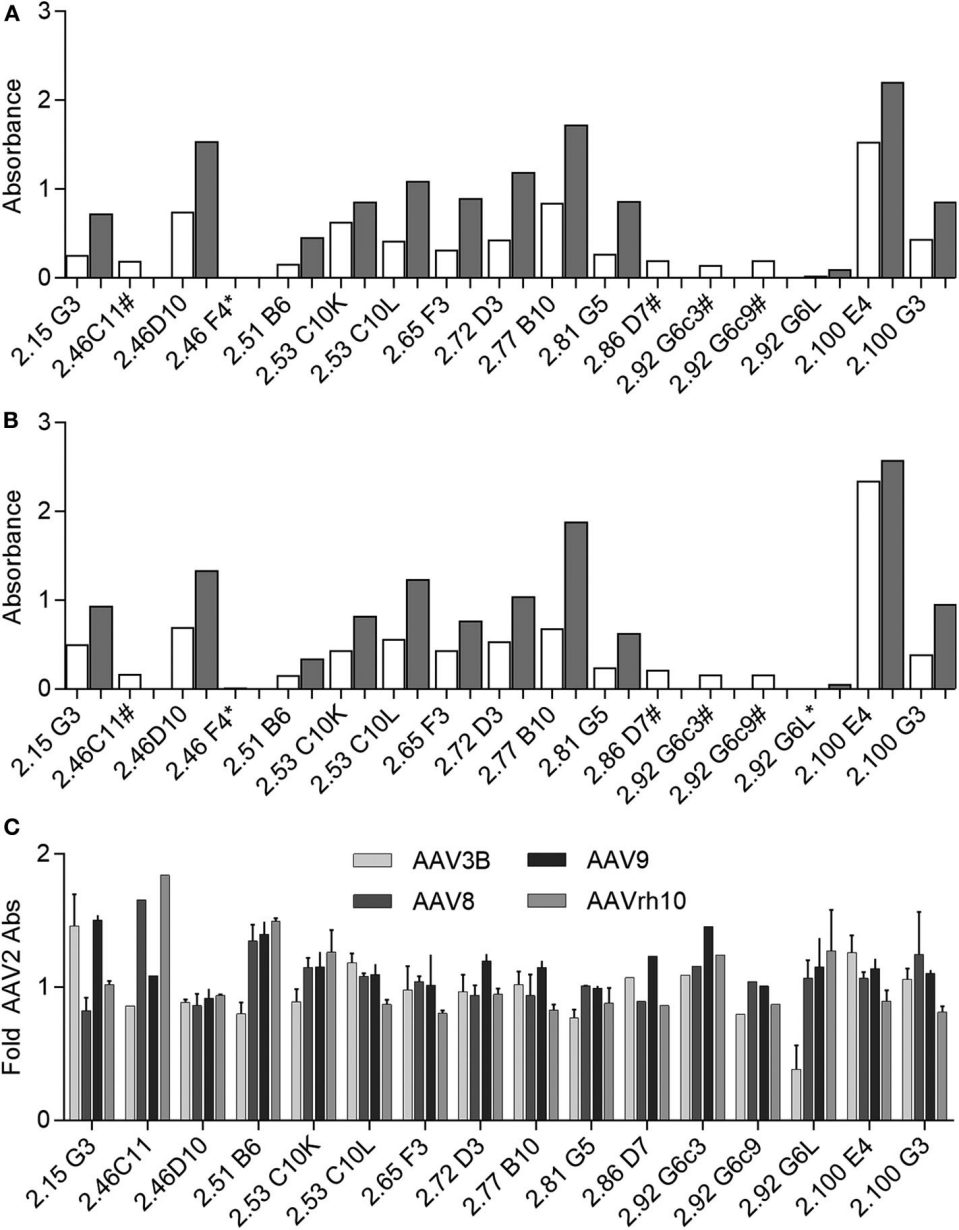
Evaluating human αAAV mAbs for AAV capsid binding. **(A,B)** Recombinant Donor 7 mAbs were produced and evaluated for **(A)** AAV2 or **(B)** AAV3B binding by ELISA. Samples were diluted to 100 ng/mL (white bars) or 250 ng/mL (gray bars) for evaluation. Some samples could not be produced at sufficient concentration to evaluate at 250 ng/mL (#); at either concentration, AAV binding could not be detected for some mAbs (*); *n* = 1 for each antibody concentration. **(C)** Recombinant mAbs (100 ng/mL or 100 ng/mL and 250 ng/mL, where applicable) were evaluated for binding to a larger panel of AAV serotypes. Binding relative to AAV2 was determined based on absorbance; *n* = 2. Data from samples for which 100 ng/mL and 250 ng/mL concentrations could be evaluated are displayed as mean ± SD.

Following cloning and *in vitro* production, we confirmed mAb recognition of AAV as well as relative binding to AAV2 as compared to AAV3B by ELISA at two concentrations: 100 ng/mL and 250 ng/mL (Figures 6A,B). Of this panel of supernatants containing recombinant mAb, 2.46F4, and 2.86D7 were evaluated next, as we were unable to produce sufficient quantities of mAb to evaluate at 100 ng/mL. Additionally, we evaluated 2.46C11, 2.92G6c3, and 2.92G6c9 exclusively at 100 ng/mL, as they did not express sufficiently to be evaluated at 250 ng/mL. All clones, with the exception of 46F4, recognized both AAV2 and AAV3B, irrespective of the serotype against which the B-cell supernatant screened positive (AAV2 only: 2.53C10, 2.65F3, 2.86D7, 2.92G6, 2.100G3; AAV3B only: 2.46C11, 2.46F4, 2.51B6, 2.77B10, 2.81G5; AAV2 and AAV3B: 2.15G3, 2.46D10, 2.72D3, 2.100E4). We were unable to detect 2.46F4 binding to either AAV2 or AAV3B capsid, indicating the need for higher concentrations for detectable binding, or that its identification was a false positive.

As we found all mAbs for which binding was detectable to be cross-reactive, we additionally evaluated binding to AAV8, AAV9, and AAVrh10, as we had determined donor 7 to be NAb positive for these three serotypes. We repeated these experiments at 100 ng/mL and 250 ng/mL, when possible. All donor 7 mAbs with detectable binding bound all serotypes tested. In order to determine capsid binding preferences, we then determined binding for each serotype relative to binding to AAV2 (Figure 6C). We found that some mAbs, including 2.46D10 and 2.81G5, bound all serotypes similarly, while other mAbs, such as 2.15G3 and 2.51B6, bound one or two serotypes preferentially. The variation in relative binding profiles suggests that while the isolated antibodies are all cross-reactive, they represent a diverse population of mAbs, likely with different binding sites. Despite having validated mAb binding to AAV, we found that none of these recombinant mAbs, either raw supernatant or purified sample, were capable of neutralizing AAV transduction *in vitro*. In sum, we have identified a panel of cross-reactive binding mAbs against AAV.

After identifying this panel of mAbs, we tried to map their epitopes by traditional cryo-reconstruction. After generating Fabs from purified, recombinant mAb, we tried optimizing complexing conditions with AAV3B, but were not fully successful. These mAbs may not be stable as Fabs and therefore are not suitable for complexing; utilization of F(ab')s instead may provide an alternate option, as was the case for AAV2 and A20 (21). Additionally, we may also utilize peptide competition assays for epitope mapping, as we found that all mAbs likely recognize linear epitopes.

## Discussion

Here, we leveraged single B-cell cloning technology to engineer permissive viral vectors that evade pre-existing immune responses. In doing so, we successfully isolated the first fully human immunoglobulins directed against our viral vector of interest, AAV. We also determined that the frequency of memory B cells against AAV2 and AAV3B in this individual donor was ~2–5%. This value is within the range reported for some viral pathogens, but is higher than the range reported for others. Importantly, this is an aspect of the pre-existing humoral immune response to AAV in humans that was previously uncharacterized. At this frequency, this approach has the potential to identify more novel human anti-AAV antibodies in a high-throughput fashion with minimal optimization (25, 56–58).

### Requirement for the Isolation of Novel NAbs

We did not identify an anti-AAV NAb in this pilot screen, and doing so would require additional isolation experiments by cloning from this donor or from a larger cohort. This suggests that in normal donors with modest neutralizing titers, the frequency of memory B cells producing NAbs is relatively low compared to those producing simply binding antibodies. Mapping neutralizing epitopes is highly useful for engineering novel AAV escape variants in order to address concerns over (1) individuals with pre-existing neutralizing titers and (2) individuals with titers induced by vector administration. However, we can still learn about the interactions of AAV with the immune system through investigating epitopes that confer antibody binding but not neutralization. Although the presence of binding antibody titers is used as an exclusion criterion in the clinic for a number of AAV gene therapy protocols (13, 59), particularly those utilizing intravenous vector administration, better characterization of these antibodies and their epitopes is still of significant value.

### Single-Cell Antibody Cloning as a Technique to Study AAV Immune Repertoires

In addition to isolating novel human mAbs against AAV, this approach will allow us to compare the immune repertoires generated in response to AAV infection or vector administration between individuals or even between routes of exposure. Many vector engineering programs assume that their escape variants will be sufficient to address immunity in the majority of individuals, despite evidence to the contrary. In particular, previous peptide competition studies of sera from individuals who are seropositive for AAV2 failed to identify even a single peptide that corresponded to neutralizing in those samples (17). Furthermore, these studies found that these individuals could be grouped into one of a number of peptide-binding profiles, completely independent of neutralizing ability (17). Additional studies in which peptides were inserted at a number of sites on AAV2 that were predicted to correspond to neutralizing epitopes revealed significant diversity in the antibody profiles of a sample panel from 29 donors (60). Taken together, these studies demonstrate that there is significant variation in the AAV antibody repertoires between individuals, which warrants further studies to inform future engineering efforts. If we are able to organize potential vector recipients into a small number of subgroups based on shared epitopes, it may be reasonable to design a variant for each subgroup. In contrast, designing a variant for each individual epitope profile may not be practical or sustainable.

Studying potential differences in the repertoire of antibodies generated following AAV infection and in response to AAV vector administration is also warranted. Historically, samples from individuals who have received vector have been limited; however, given the recent rise of AAV use in the clinic, these studies are now possible. Additionally, researchers can evaluate potential differences in the humoral immune response due to route of administration and natural vs. recombinant vector-induced immunity using this approach. In addition to identifying shared and divergent antibody epitopes, sequence analysis of the immunoglobulin genes of the B-cell populations in each group may allow a better understanding of how AAV uniquely interacts with the immune system as a replication-deficient, non-pathogenic virus, and how that interaction changes when AAV is transformed into a recombinant viral vector.

In conclusion, these studies demonstrate the application of B-cell cloning technologies to the field of gene therapy. Despite our initial failed attempts at epitope mapping, this work shows the potential of this approach to not only help identify and evaluate novel anti-AAV antibodies, but also use those antibodies to study how AAV interacts with the immune system in different patient populations. We hope to utilize this approach to better understand basic AAV biology as it pertains to the immune response and to use that information to design the next generation of AAV therapeutics.

## Data Availability Statement

All datasets presented in this study are included in the article/Supplementary Material.

## Author Contributions

AG, AT, and JW were responsible for conceptualization. AG, RC, and AT were responsible for experimental design. AG was responsible for experimental execution, data analysis, and manuscript composition. All authors contributed to the article and approved the submitted version.

## Conflict of Interest

JW is a paid advisor to and holds equity in Scout Bio and Passage Bio. He holds equity in Surmount Bio. He also has a sponsored research agreement with Ultragenyx, Biogen, Janssen, Precision Biosciences, Moderna Inc., Scout Bio, Passage Bio, Amicus Therapeutics, and Surmount Bio, which are licensees of Penn technology. JW is an inventor on patents that have been licensed to various biopharmaceutical companies and for which he may receive payments. AT is an inventor on patents that have been licensed to various biopharmaceutical companies. AG is an inventor on patents that may be licensed to various biopharmaceutical companies and may receive royalties. The remaining author declares that the research was conducted in the absence of any commercial or financial relationships that could be construed as a potential conflict of interest.

## Supplementary Material

Supplementary Figure 1 Representative flow cytometry data of donor B cell populations, pre- and post-bead sort. Cells were first gated on CD19 status, and positive cells were evaluated for the presence of CD27 and IgM.

Supplementary Figure 2 Phylogeny of isolated variable heavy chain sequences. Phylogenies of **(A)** validated and **(B)** total isolated variable heavy chain sequences. Clones and germline V gene usage are indicated. Figure was generated in AlignX.

Supplementary Figure 3 Phylogeny of isolated variable light chain sequences. Phylogenies of **(A)** validated kappa, **(B)** total kappa, **(C)** validated lambda, and **(D)** total lambda isolated variable heavy chain sequences. Clones and germline V gene usage are indicated. Figure was generated in AlignX.
