# Supplementary figures and images for: Isolating Human Monoclonal Antibodies Against Adeno-Associated Virus From Donors With Pre-existing Immunity
Source: Front Immunol. 2020 Jul 7;11:1135. doi: 10.3389/fimmu.2020.01135 (PMC7358261; doi:10.3389/fimmu.2020.01135)

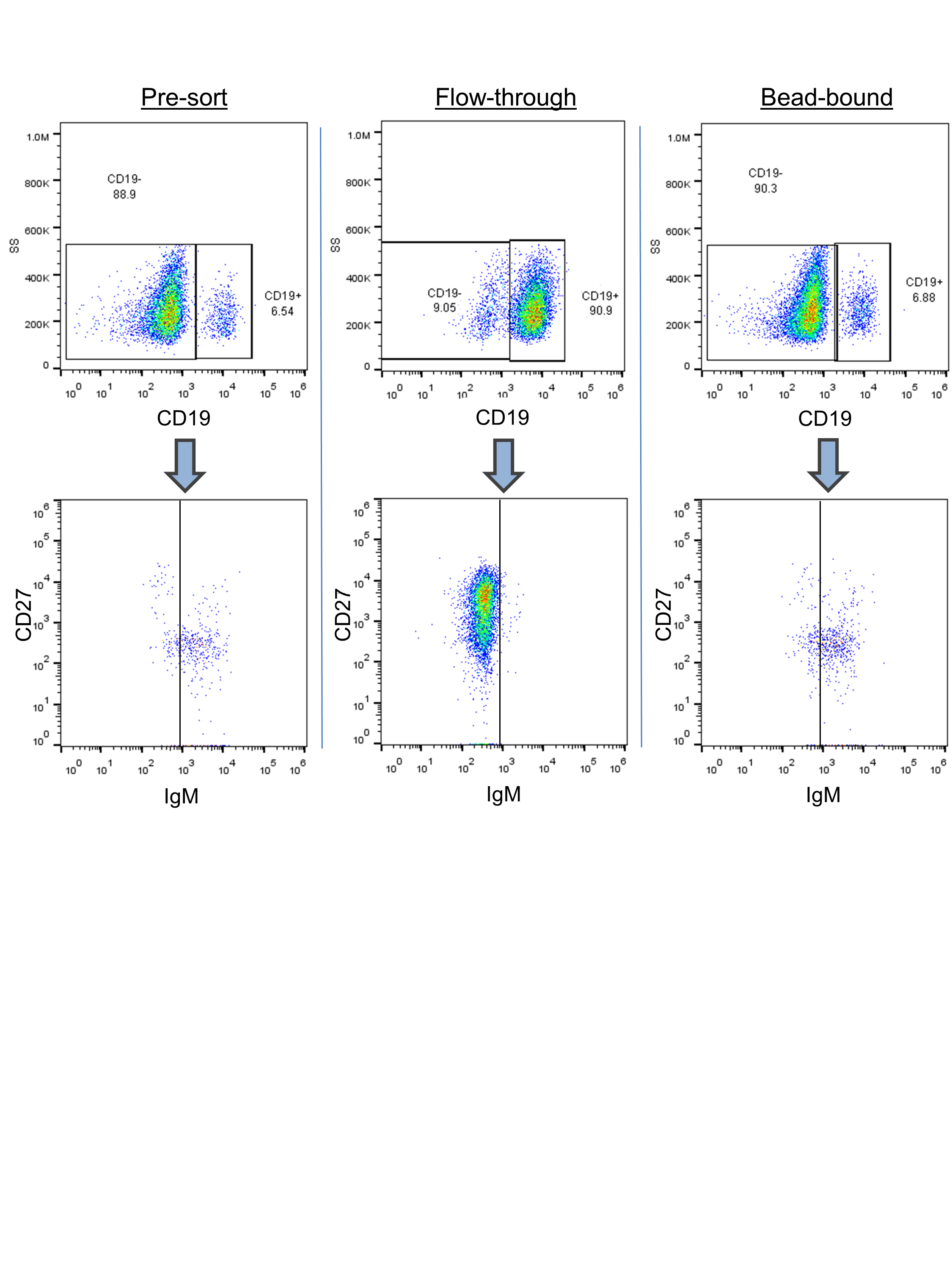

Supplement: Supplementary Figure 1 — Representative flow cytometry data of donor B cell populations, pre- and post-bead sort. Cells were first gated on CD19 status, and positive cells were evaluated for the presence of CD27 and IgM. [file Image_1.TIF]

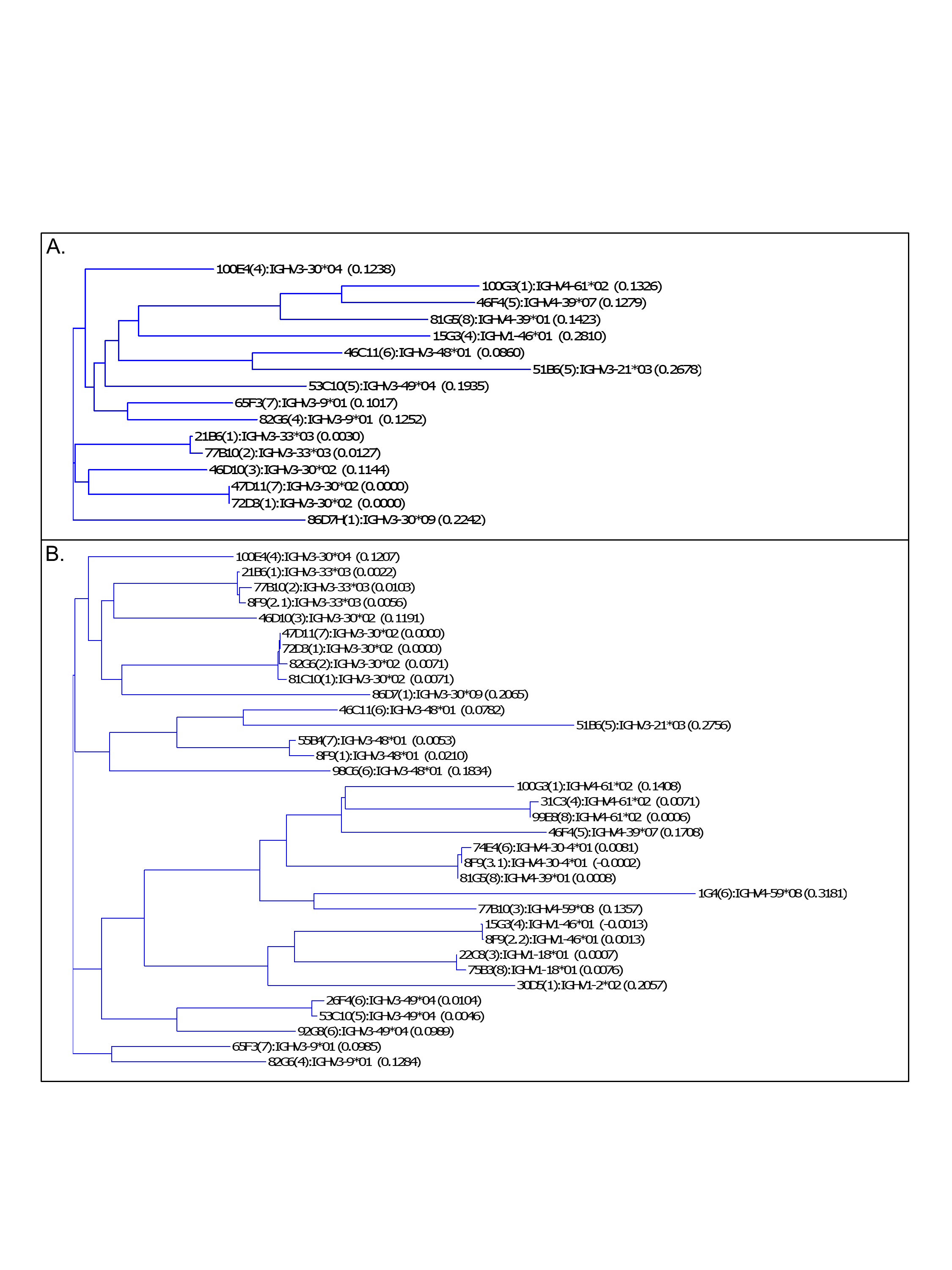

Supplement: Supplementary Figure 2 — Phylogeny of isolated variable heavy chain sequences. Phylogenies of (A) validated and (B) total isolated variable heavy chain sequences. Clones and germline V gene usage are indicated. Figure was generated in AlignX. [file Image_2.TIF]

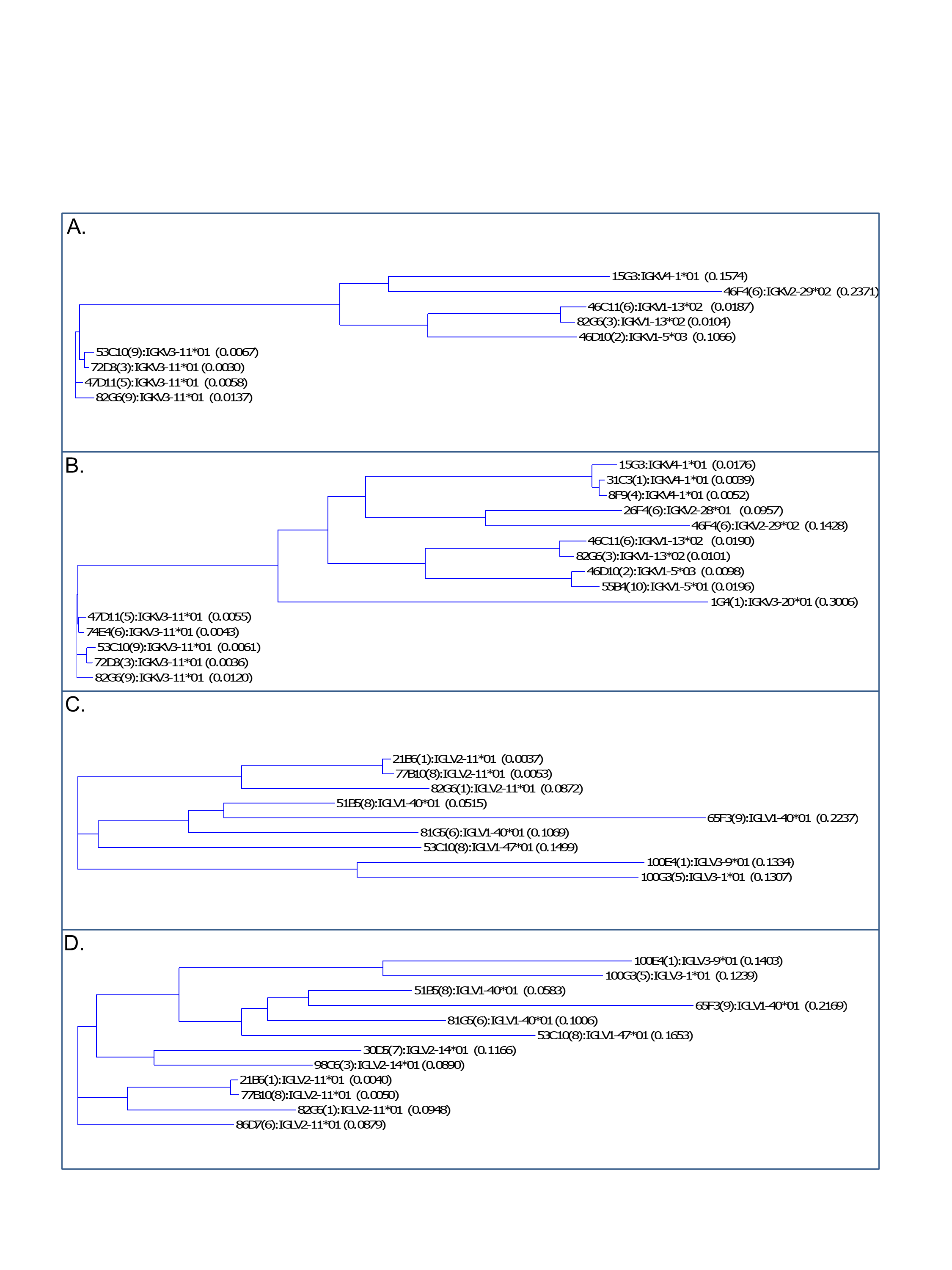

Supplement: Supplementary Figure 3 — Phylogeny of isolated variable light chain sequences. Phylogenies of (A) validated kappa, (B) total kappa, (C) validated lambda, and (D) total lambda isolated variable heavy chain sequences. Clones and germline V gene usage are indicated. Figure was generated in AlignX. [file Image_3.TIF]
